# Primary extragastrointestinal stromal tumour of the whole abdominal cavity, omentum, peritoneum and mesentery: a case report and review of the literature

**DOI:** 10.1186/1752-1947-8-337

**Published:** 2014-10-10

**Authors:** Nassir Alhaboob Arabi, Abdulmagid M Musaad, Elsaggad Eltayeb Ahmed, Abdulmunem A Abdo, Ahmed M Elhassan, Hiba Hassan, Nasreeldeen Adam, Mohamed Abdelazeem, Mohamed A Ibnouf

**Affiliations:** 1 Department of GI Surgery, Ibn Sina hospital, Khartoum, Sudan; 2 Department of GI Surgery, Fedail Hospital, Khartoum, Sudan; 3 Department of Gastroenterology, Ibn Sina Hospital, Khartoum, Sudan; 4 Department of Pathology, University of Khartoum, Khartoum, Sudan; 5 Department of Surgery, Shandi Hospital, Shandi, Sudan; 6 Department of Surgery, Ibn Sina Hospital, Fedail Hospital, Khartoum, Sudan

**Keywords:** Abdominal mass, EGIST, Extragastrointestinal stromal tumour, GIST, Nodules

## Abstract

**Introduction:**

The gastrointestinal stromal tumour is one of the common mesenchymal tumours of the gastrointestinal tract. It originates from the interstitial cells of Cajal. Gastrointestinal stromal tumours that present outside the gastrointestinal tract are called extragastrointestinal stromal tumours; they share the same morphological and immunohistochemical characteristics. Here we describe an unusual case of extragastrointestinal stromal tumour that presented with gooseberry-like multiple nodules involving the whole abdominal cavity.

**Case presentation:**

A 65-year-old Sudanese man presented with vague abdominal pain and progressive abdominal distension for 6 months. The pain was associated with mild loss of weight despite good appetite. A physical examination revealed distended abdomen with multiple firm nodules involving his whole abdomen. The results of haematological tests were within normal range. Ultrasound of his abdomen showed multiple nodules of varying sizes in the peritoneal cavity. A computed tomography scan of his abdomen showed numerous nodules of different sizes (1 to 3cm in diameter) filling the whole peritoneal cavity with intense peripheral enhancement. Ultrasound-guided biopsy was not informative. Upper and lower gastrointestinal endoscopies were normal. Exploration of his abdomen revealed multiple firm gooseberry-like nodules of different sizes involving the greater omentum, peritoneal cavity and the mesentery. The liver, spleen and pancreas were normal. The result of the histopathology was conclusive for gastrointestinal stromal tumour.

**Conclusions:**

Here we present a rare case of extragastrointestinal stromal tumour in a patient who presented with vague abdominal pain and progressive abdominal distension. A laparotomy showed gooseberry-like multiple nodules of different sizes involving his whole abdominal cavity. He underwent debulking surgery and received imatinib.

## Introduction

Gastrointestinal stromal tumours (GISTs) comprise 1 to 3% of all gastrointestinal malignancies. They are typically defined as tumours whose behaviour is driven by mutations in the *KIT* gene or *PDGFRA* gene, and may or may not stain positively for *KIT* gene [1]. Due to the presence of tyrosine kinase receptors within the tumour tissue, GIST is thought to originate from gastrointestinal pacemaker cells, the interstitial cells of Cajal (ICC). Sometimes tumours with the same morphological and immunohistochemical characteristics as GISTs are detected outside the alimentary canal, hence they are called extragastrointestinal stromal tumours (EGISTs). The biological behaviour of these tumours is uncertain and the malignancy rates are difficult to predict [2]. Here we present an unusual case of EGIST that presented with multiple gooseberry-like nodules involving the whole abdominal cavity, the omentum, peritoneum and small bowel mesentery, which makes a radical resection difficult.

## Case presentation

**Figure 1:**
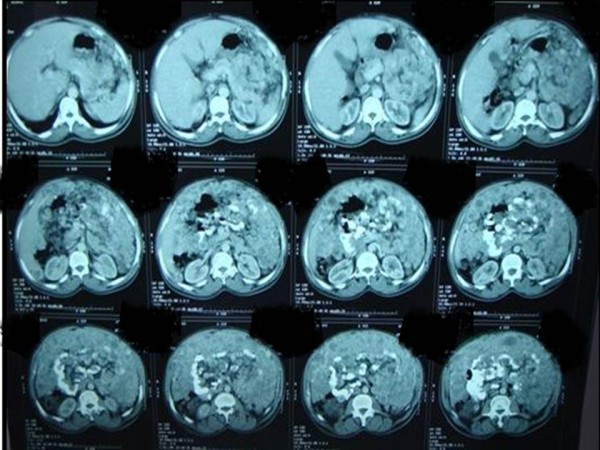
A computed tomography scan of abdomen showed numerous nodules of different sizes.

**Figure 2:**
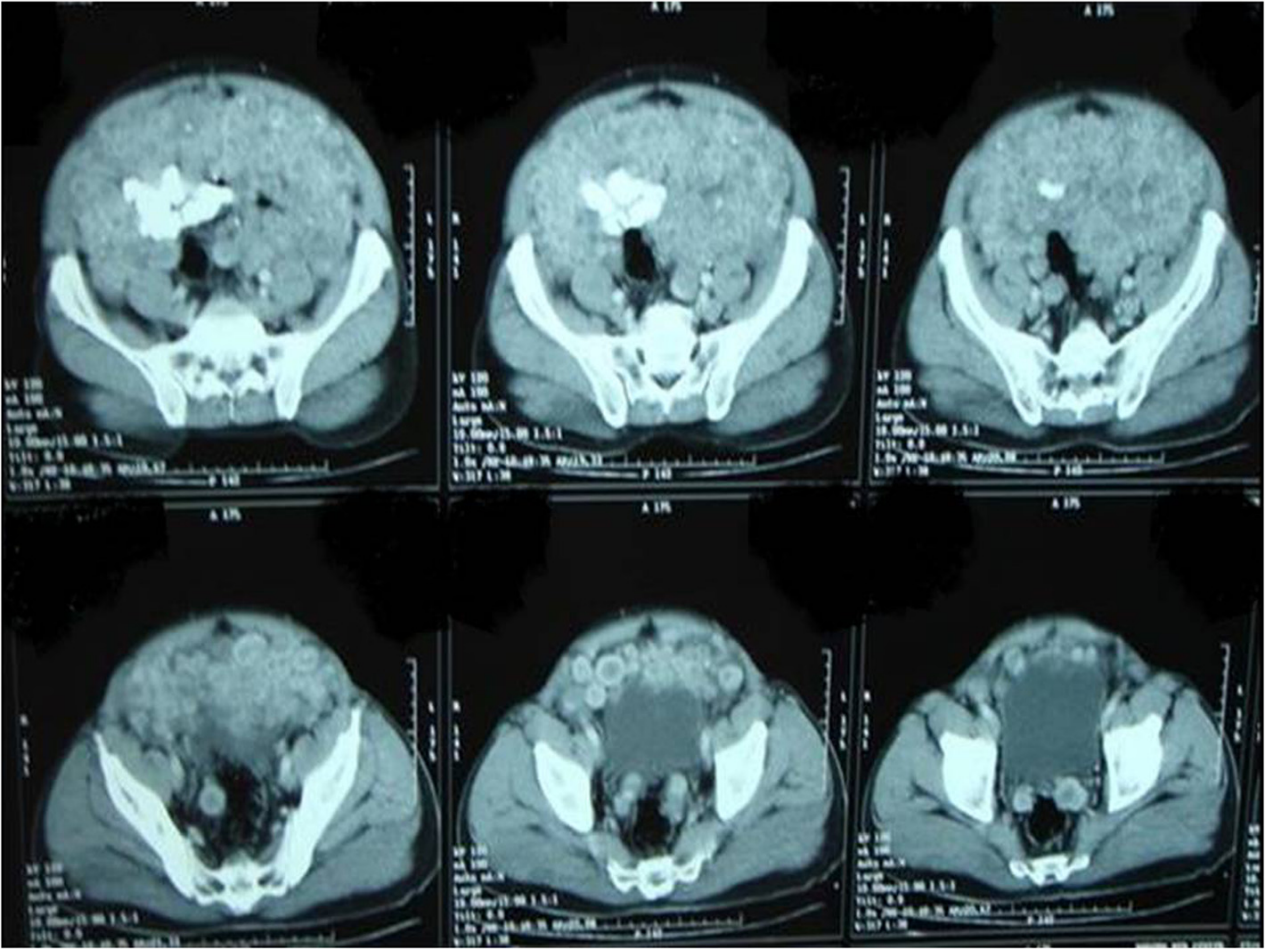
A computed tomography scan showed numerous nodules of different sizes (pelvis view).

**Figure 3:**
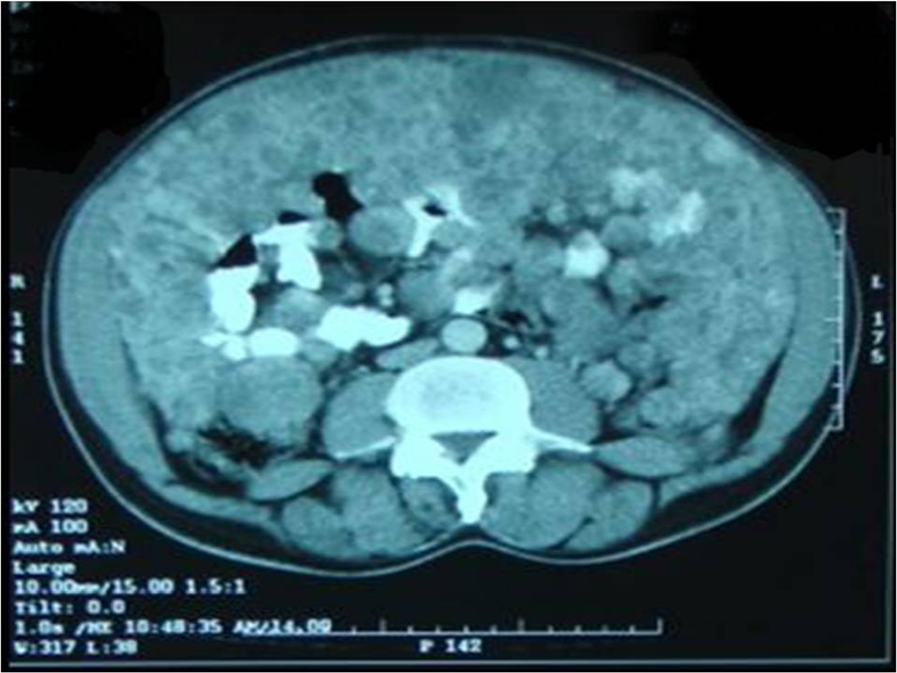
A computed tomography scan of abdomen showed numerous nodules filling the peritoneal cavity and the surrounding bowel loops.

**Figure 4:**
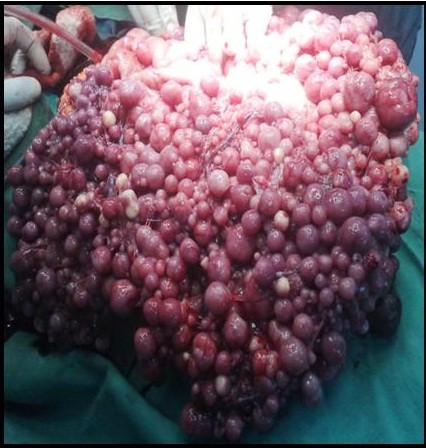
Intra-operative picture showed multiple firm gooseberry-like nodules between the bowel loop.

**Figure 5:**
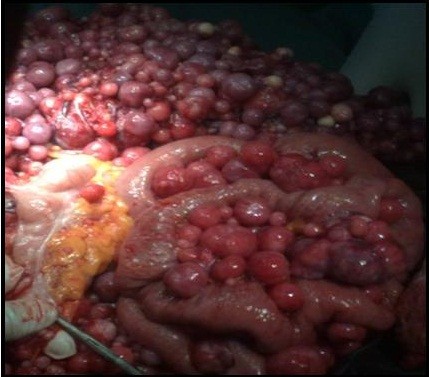
Intra-operative picture showed multiple firm gooseberry-like nodules involving the greater omentum, peritoneal cavity and the mesentery.

**Figure 6:**
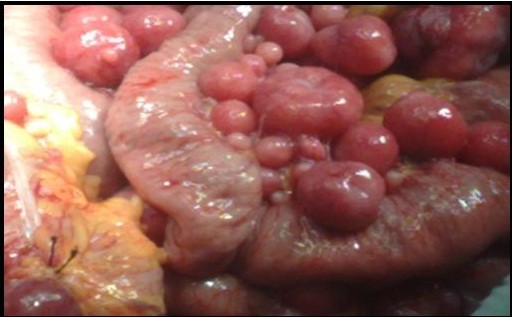
Intra-operative picture showed attachment of the nodules to the bowel loop.

**Figure 7:**
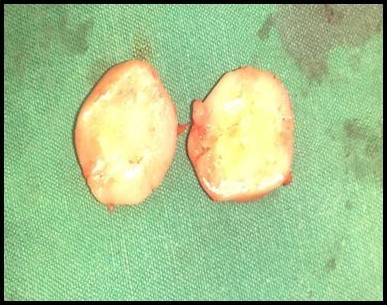
Morphological picture of single nodule.

A 65-year-old Sudanese man, who was previously well, presented with vague central abdominal pain. The pain was increasing gradually. It was constant, associated with progressive abdominal distension for the past 6 months and mild loss of weight despite good appetite. A physical examination revealed distended abdomen with multiple firm nodules in his abdomen. Liver and spleen were not palpable. The results of haematological tests were within normal range; an ultrasound of his abdomen revealed multiple nodules of varying sizes in the peritoneal cavity. A computed tomography scan of his abdomen showed numerous nodules of different sizes, 1 to 3cm in diameter each, filling the peritoneal cavity and the surrounding bowel loops with intense peripheral enhancement (Figures 1, 2 and 3). Ultrasound-guided biopsy was not conclusive. Gastroscopy and colonoscopy showed normal stomach and colon. Exploration revealed multiple firm gooseberry-like nodules of different sizes ranging between 1 and 5cm in diameter (Figures 4, 5, 6 and 7), involving the greater omentum, peritoneal cavity and the mesentery, but liver texture was normal. The main bulk of the tumour was excised together with the greater omentum and part of the mesentery, however, residual tumour remained stuck to the small bowel and great vessels. The postoperative period was uneventful, and he was discharged 5 days later. Histopathology reported presence of sheets of cellular tumour composed of spindle cells infiltrating smooth muscle fibres, with positive CD117 stain. Hence the diagnosis of GIST was made. The patient was then referred to an oncologist and received imatinib but with little improvement in his symptoms.

## Discussion

GISTs are uncommon tumours of the gastrointestinal tract (GIT). They originate from ICC in the stomach, but they can appear anywhere along the GIT. GISTs rarely occur outside the alimentary canal; hence, those that do are called EGISTs. The behaviour of GISTs ranges from benign to cancerous. Bülbül Doğusoy studied 1160 cases of GISTs. He reported a male-to-female ratio of 1.22 and a mean age of 56.75 years. He found the stomach to be the most common location (45.0%), followed by the small intestine (32.0%), omentum-peritoneum (12.6%), large intestine (9.3%), and oesophagus (1.1%) [3]. Miettinen *et al.* analysed 95 patients with GISTs designated as omental masses in 49 males and 46 females, with a median age of 60 (range: 27 to 88) years. This tumour was found as a single mass in 51 patients, and as multiple masses in 39 patients. He added that omental GISTs unattached to the alimentary canal often resemble gastric GISTs and multiple omental GISTs often resemble small intestinal GISTs suggesting that they may be metastatic [4]. Reith *et al.* reported that the majority of EGISTs are large, that is <10cm in diameter, when first detected, whereas small (and presumably early) EGISTs are rarely encountered because they seldom produce symptoms. Two of their four cases were smaller than 5cm and detected during unrelated workup [5]. Genetically, EGISTs express CD117 (c-kit receptor; 100%), CD34 (50%), neuron-specific enolase (44%), smooth muscle actin (26%), desmin (4%), and S-100 protein (4%) [5]. The clinical, pathological and prognostic features of GISTs are widely known, whereas data about EGISTs are very few and the incidence, histogenesis and histological predictors of outcome are not yet defined [6]. Many studies have been done to identify the origin of EGIST, Miettinen and Lasota reported that omental and mesenteric EGISTs are derived from stomach and small intestine respectively, representing tumours that, for some reason, have detached from their gastrointestinal original site during their development [7]. However, Reith *et al.* reported that extragastrointestinal soft tissue stromal tumours are histologically and immunophenotypically similar to their gastrointestinal counterpart, but EGISTs have an aggressive course more akin to small intestinal than gastric stromal tumours [5]. There are many questions about the association between GIST and EGIST. AbdullGaffar showed that the association between non-incidental GISTs and extra-GIT tumours is difficult to determine in the majority of cases. This association is most probably a coincidental finding. AbdullGaffar reported a case series of possible association of GISTs with extra-GIT tumours in female patients and, like other studies, AbdullGaffar suggested that patients, especially women, with GISTs should be investigated and followed up for the possibility of coexisting GIT and extra-GIT neoplasms [8].

Regarding the prognosis in relation to the site of origin, a study of more than 1000 cases of GIST subdivided into five locations (oesophagus, stomach, small and large bowels, versus peritoneum, mesentery, and omentum) found that the tumour site had an independent prognostic factor. Oesophageal tumours had the most favourable prognosis, whereas peritoneal tumours had the lowest survival rate [9]. This seems to be due to the early diagnosis of oesophageal GISTs related to the early appearance of symptoms. By contrast, in the other sites, especially the abdominal cavity, patients had slow onset of disease and symptoms remained vague until the tumour became large in size.

Despite significant advances in new chemotherapeutic drugs, radical surgery remains the only method for long-term survival. Although further data are required to evaluate its use in the adjuvant and neoadjuvant settings, imatinib mesylate currently provides the most effective treatment option in the management of advanced cases [10]. In our case complete surgical tumour resection remains a dilemma because it was extremely difficult to remove the whole nodules and the only option that remained was imatinib.

## Conclusions

Our case was rare case of EGIST in a man who presented with vague abdominal pain and progressive abdominal distension. Exploration revealed multiple gooseberry-like nodules of different sizes that involved the whole abdominal cavity. Radical excision was not possible. On histopathology the tumour was CD117 positive. The patient underwent debulking surgery and received imatinib with little improvement in his symptoms.

## Consent

Written informed consent was obtained from the patient for publication of this case report and accompanying images. A copy of the written consent is available for review by the Editor-in-Chief of this journal.

## Abbreviations

EGIST: Extragastrointestinal stromal tumour; GIST: Gastrointestinal stromal tumour; GIT: Gastrointestinal tract; ICC: Interstitial cells of Cajal.

## Competing interests

The authors declare that they have no competing interests.

## Authors’ contributions

AA and HH admitted the patient and requested the relative investigations. AM, EEA, NA, and MA performed the surgery and the postoperative follow up. AE processed the histopathology and its report. NA wrote the manuscript. MI participated in its design and coordination and helped to draft the manuscript and reviewed the paper for English editing. All authors read and approved the final manuscript.

## References

[B1] Miettinen M Lasota J Gastrointestinal stromal tumours: review on morphology, molecular pathology, prognosis, and differential diagnosis Arch Pathol Lab Med 2006 130 10 1466 1478 doi:10.1043/1543 17090188 10.5858/2006-130-1466-GSTROM

[B2] Kolarík J Drápela J Extragastrointestinal stromal tumour (EGIST) – a case review Rozhl Chir 2012 91 4 241 245 22880273

[B3] Turkish GIST Working Group Bülbül Doğusoy G Gastrointestinal stromal tumours: a multicentre study of 1160 Turkish cases Turk J Gastroenterol 2012 23 3 203 211 22798108

[B4] Miettinen M Sobin LH Lasota J Gastrointestinal stromal tumours presenting as omental masses – a clinicopathologic analysis of 95 cases Am J Surg Pathol 2009 33 9 1267 1275 doi:10.1097/PAS.0b013e3181a13e99 10.1097/PAS.0b013e3181a13e99 19440146

[B5] Reith JD Goldblum JR Lyles RH Weiss SW Extragastrointestinal (soft tissue) stromal tumors: an analysis of 48 cases with emphasis on histologic predictors of outcome Mod Pathol 2000 13 5 577 10.1038/modpathol.3880099 10824931

[B6] Franzini C Alessandri L Piscioli I Donato S Faraci R Morelli L Del Nonno F Licci S Extra-gastrointestinal stromal tumour of the greater omentum: report of a case and review of the literature World J Surg Oncol 2008 6 25 doi:10.1186/1477-7819-6-25 10.1186/1477-7819-6-25 18294396 PMC2267191

[B7] Miettinen M Lasota J Gastrointestinal stromal tumours – definition, clinical, histological, immunohistochemical, and molecular genetic features and differential diagnosis Virchows Arch 2001 438 1 12 10.1007/s004280000338 11213830

[B8] AbdullGaffar B Gastrointestinal stromal tumours and extra-gastrointestinal tract neoplasms South Med J 2010 103 10 1004 1008 10.1097/SMJ.0b013e3181ef2f41 20818304

[B9] Emory TS Sobin LH Lukes L Lee DH O’Leary TJ Prognosis of gastrointestinal smooth-muscle (stromal) tumours: dependence on anatomic site Am J Surg Pathol 1999 23 82 87 10.1097/00000478-199901000-00009 9888707

[B10] Barghash IA Abdul-Samad M Oteifa M Adesina AO Gastrointestinal stromal tumour of the omentum: a case report Gulf J Oncolog 2008 3 58 63 20084799

